# Editorial: Dopaminoceptive forebrain regions: a search for structural and functional organization underlying normal and impaired social adaptation

**DOI:** 10.3389/fnana.2025.1694730

**Published:** 2025-09-22

**Authors:** Loreta Medina, András Csillag

**Affiliations:** ^1^Department of Experimental Medicine, Universitat de Lleida, Lleida, Spain; ^2^Institut de Recerca Biomèdica de Lleida-Fundació Privada Dr. Pifarré (IRBLleida), Lleida, Spain; ^3^Department of Anatomy, Histology and Embryology, Semmelweis University, Budapest, Hungary

**Keywords:** prosomeric model, dopamine receptor, tyrosine hydroxylase, extended amygdala, striatum, hypothalamus, Otp cells, valproic acid

This Research Topic explored the structural and functional organization of forebrain dopaminergic (DAergic) systems. These systems encompass multiple cell groups and pathways involved in reward, motivation, motor control, homeostasis, and social behavior modulation. It comprises six articles spanning various vertebrate species, addressing novel aspects of DAergic cell group evolution, development, and neuromeric organization, along with functions of specific mesocortical and olfactory subsystems along with alterations due to gestational valproic acid (VPA) exposure that lead to autism-like traits.

The ventral tegmental area (VTA) and the substantia nigra (SN) are the most studied DAergic groups in mammals and other amniotes due to their critical roles in reward and motor control (Smeets and Reiner, 1994; Björklund and Dunnett, 2007). While these are classically viewed as midbrain structures, developmental research suggests a segmental organization involving both diencephalic and mesencephalic origins (Puelles and Medina, 1994). Ferran et al. used the updated prosomeric model to examine the segmental origin of VTA and SN DAergic neurons during development in rodents, non-human primates, and humans. By combining tyrosine hydroxylase (TH) immunohistochemistry with gene expression and morphological landmarks, they identified six segments (five in the forebrain and one in the isthmic r0) giving rise to these neurons. Such multi-neuromeric origin may underlie functional specialization, and different connectivity and target patterns, with potential implications for vulnerability to degeneration, hypoxia, and neurodevelopmental disorders.

VTA and SN DAergic neurons project to the telencephalon, targeting the striatum and, via mesocortical and mesolimbic pathways, the cortex and extended amygdala (Reiner et al., 1998; Björklund and Dunnett, 2007). Messore et al. focused on cortical dopamine modulation by manipulating layer 6 neurons expressing dopamine receptor 1 in the mouse somatosensory cortex. These cells are considered remnants of the subplate, which is essential for the establishment of thalamocortical connections. Messore et al. demonstrated that these dopaminoceptive cells also play a critical role in the activation of cortico-thalamo-cortical loops, possibly participating in stimulus representation and sensory processing.

In tetrapods, the majority of DAergic axons targeting the striatum and pallium arise from the perikarya of the VTA and/or SN (Smeets and González, 2000; except in the olfactory bulb, which contains intrinsic DAergic neurons in all vertebrates). In fish, however, at least part of the telencephalic innervation comes from locally-born DAergic neurons (Rodríguez-Moldes et al.). The presence of these cells in the telencephalon was once thought to be specific for fish, but minor subsets of catecholaminergic (CAergic) cells were later found in the cerebral cortex/pallium and/or the striatum of mammals and birds (Marín et al., 2005; Bupesh et al., 2014; Fujita et al.). Tracing the evolution of dopaminoceptive regions of the telencephalon, Rodríguez-Moldes et al. studied the development of area superficialis basalis in the catshark (a cartilaginous fish closely related to ancestral jawed vertebrates), using neuronal phenotypic markers and transcription factors of the pallium or the subpallium. The authors showed that this area is subpallial with cells derived from striatal and pallidal subdivisions; however, it becomes quite complex due to tangential migrations during development. In catsharks, some cells from the pallidal embryonic domain migrate into the striatum, giving rise to an ectopic globus pallidus that resembles the situation observed in birds. Rodríguez-Moldes et al. suggested that the area superficialis basalis of Chondrychtians contains precursor cells for the striatum, pallidum, and extended amygdala, which may represent a forerunner of these regions in later tetrapods, opening new avenues for research.

In vertebrates, the medial extended amygdala and several preoptic and hypothalamic centers that are involved in the modulation of social behavior and/or homeostasis also contain subpopulations of CAergic neurons (Bupesh et al., 2014; Vicario et al., 2014; Smeets and González, 2000; Fujita et al.). These cells are morphologically and functionally diverse. Some of them are DAergic (expressing TH and the aromatic amino acid decarboxylase enzyme), while others only express TH (Ahmed et al., 2012; Ugrumov, 2024). The heterogeneity and manifold functions of these CAergic cells reflect their origin from molecularly distinct progenitors (Romanov et al., 2020), located in different embryonic divisions with precise topological locations along the rostrocaudal (neuromeric) and dorsoventral axes of the forebrain (Puelles and Medina, 1994; Bilbao et al., 2022; Ferran et al.). Using Otp-eGFP mice, Morales et al. showed that some subsets of CAergic neurons in the extended amygdala, preoptic area and different nuclei of the hypothalamus derive from cells that express the transcription factor Otp. However, these centers also contain non-Otp CAergic neurons that appear to derive from different progenitors that express other transcription factors (Romanov et al., 2020; Zhang et al., 2021), raising questions on the function of different Otp and non-Otp CAergic neurons in the modulation of social behavior and homeostasis.

In vertebrates, olfactory information that reaches the medial extended amygdala directly or indirectly plays a critical role in social behavior. In this Topic, Fujita et al. showed that the main projection neurons of the olfactory bulb in chickens, including mitral cells, selectively express the dopamine receptor 4 (DRD4), indicating that their activity is modulated by dopamine through this specific receptor. In mammals and birds, DRD4 polymorphisms are associated with animal personality traits, a feature that contributes to the shaping of social behavior. The authors' findings prompt further investigation into the role of dopaminoceptive mitral cells of the olfactory bulb in relation to personality and social behavior.

CAergic systems start to form early in development, and they appear to act as morphogens, modulating axonal growth and the phenotype of target cells (Ugrumov, 2024). The study by Finszter et al. pointed out that DAergic projections may determine the formation of synaptic patterns in target regions during development. Following gestational VPA treatment of mice (leading to autistic-like traits), an overall reduction in DA input to the ventral striatum (olfactory tubercle, OT; nucleus accumbens, NAc) was observed, together with a decrease in DAergic synaptic contacts with calbindin (in the OT) or calretinin (in the NAc) expressing interneurons. These findings indicate that the shaping of the social and reward systems is affected by prenatal VPA exposure through alteration of DAergic pathways. Future studies are needed to better understand the role of other dopaminoceptive regions, including those modulating social behavior, in autism.

Overall, the articles presented in this Research Topic improve our understanding of the evolution, development, segmental organization and function of DAergic and dopaminoceptive forebrain centers, and open new avenues for future DAergic system research.

## References

[B1] Ahmed E. I. Northcutt K. V. Lonstein J. S. (2012). L-amino acid decarboxylase- and tyrosine hydroxylase-immunoreactive cells in the extended olfactory amygdala and elsewhere in the adult prairie vole brain. J. Chem. Neuroanat. 43, 76–85. 10.1016/j.jchemneu.2011.10.006 22074805

[B2] Bilbao M. G. Garrigos D. Martinez-Morga M. Toval A. Kutsenko Y. Bautista R.. (2022). Prosomeric hypothalamic distribution of tyrosine hydroxylase positive cells in adolescent rats. Front. Neuroanat. 16:868345. 10.3389/fnana.2022.868345 35601999 PMC9121318

[B3] Björklund A. Dunnett S. B. (2007). Dopamine neuron systems in the brain: an update. Trends Neurosci. 30, 194–202. 10.1016/j.tins.2007.03.006 17408759

[B4] Bupesh M. Vicario A. Abellán A. Desfilis E. Medina L. (2014). Dynamic expression of tyrosine hydroxylase mRNA and protein in neurons of the striatum and amygdala of mice, and experimental evidence of their multiple embryonic origin. Brain Struct. Funct. 219, 751–776. 10.1007/s00429-013-0533-7 23479178 PMC4023077

[B5] Marín F. Herrero M. T. Vyas S. Puelles L. (2005). Ontogeny of tyrosine hydroxylase mRNA expression in mid- and forebrain: neuromeric pattern and novel positive regions. Dev. Dyn. 234, 709–717. 10.1002/dvdy.20467 15973733

[B6] Puelles L. Medina L. (1994). “Development of neurons expressing tyrosine hydroxylase and dopamine in the chicken brain: a comparative segmental analysis,” in Phylogeny and Development of Catecholamine Systems in the CNS of Vertebrates, eds. W. J. A. J. Smeets, and A. Reiner (Cambridge: Cambridge University Press), 381–401.

[B7] Reiner A. Medina L. Veenman C. L. (1998). Structural and functional evolution of the basal ganglia in vertebrates. Brain Res. Brain Res. Rev. 28, 235–285. 10.1016/S0165-0173(98)00016-2 9858740

[B8] Romanov R. A. Tretiakov E. O. Kastriti M. E. Zupancic M. Häring M. Korchynska S.. (2020). Molecular design of hypothalamus development. Nature 582, 246–252. 10.1038/s41586-020-2266-0 32499648 PMC7292733

[B9] Smeets W. Reiner A. (Eds.) (1994). Phylogeny and Development of Catecholamine Systems in the CNS of Vertebrates. Cambridge: Cambridge University Press.

[B10] Smeets W. J. González A. (2000). Catecholamine systems in the brain of vertebrates: new perspectives through a comparative approach. Brain Res. Brain Res. Rev. 33, 308–379. 10.1016/S0165-0173(00)00034-5 11011071

[B11] Ugrumov M. V. (2024). Hypothalamic neurons fully or partially expressing the dopaminergic phenotype: development, distribution, functioning and functional significance. A review. Front. Neuroendocrinol. 75:101153. 10.1016/j.yfrne.2024.101153 39128801

[B12] Vicario A. Abellán A. Desfilis E. Medina L. (2014). Genetic identification of the central nucleus and other components of the central extended amygdala in chicken during development. *Front. Neuroanat*. 8:90. Erratum in: Front Neuroanat. 15:671725. 10.3389/fnana.2021.671725 25309337 PMC4159986

[B13] Zhang Y. H. Xu M. Shi X. Sun X. L. Mu W. Wu H.. (2021). Cascade diversification directs generation of neuronal diversity in the hypothalamus. Cell Stem Cell 28, 1483–1499.e8. 10.1016/j.stem.2021.03.020 33887179

